# Perceived abusive supervision and mental health among Chinese graduate students: the chain mediating roles of autonomy need and professional identity

**DOI:** 10.1186/s40359-025-03324-5

**Published:** 2025-08-23

**Authors:** Yingying Yao, Jianqiao Chen, Haoyang Chi, Yaming Hang, Zhihong Qiao

**Affiliations:** 1 Psychological Counselling and Education Centre, Student Affairs Department, Xiamen University, Xiamen, Fujian China; 2 Mental Health Education Centre, Student Affairs Department, University of International Business and Economics, Beijing, China; 3 Mental Health Education and Counselling Centre, Student Affairs Department, Ocean University of China, Qingdao, Shandong China; 4 Faculty of Psychology, Beijing Normal University, Beijing, China

**Keywords:** Abusive supervision, Mental health, Autonomy need, Professional identity, Supervisor-student relationship

## Abstract

**Background:**

The mental health issue of graduate students related to the strained relationships with their academic supervisors has triggered wide concern and heated discussion recently. The study aimed to explore the relationship between abusive supervision and the mental health of graduate students, and the mediating roles of autonomy need and professional identity.

**Method:**

An online survey was conducted among 233 graduate students and the perceived abusive supervision, autonomy need, professional identity and the three indicators of mental health (anxiety, depressive symptoms and psychache) were measured.

**Results:**

It was found that perceived abusive supervision positively correlated to the latent variable mental health, and it worked through the respective mediating effects of autonomy need (*β* = 0.060, *p* = 0.004, 95% *CI* = [0.019, 0.100]), professional identity (*β* = 0.054, *p* = 0.004, 95% *CI* = [0.017, 0.090]) and the chain mediating effect of them (*β* = 0.006, *p* = 0.028, 95% *CI* = [0.001, 0.011]). The total indirect effect size was 0.119 (*p* < 0.001, 95% *CI* = [0.066, 0.173]), accounting for 24.44% of overall effect size. These findings deepen the understanding of the influence of abusive supervision on mental health among graduate students and provide practical insights into psychosocial intervention from the perspective of the Conservation of Resources theory and Self-Determination theory.

**Supplementary Information:**

The online version contains supplementary material available at 10.1186/s40359-025-03324-5.

## Introduction

### Background

Throughout the graduate training, graduate students have been reported to encounter considerable stress and suffer from related negative mental health outcomes. A global survey revealed that the rates for graduate students reporting moderate to severe depression or anxiety were separately 39% and 41% which were six times higher than those in other populations [1]. For graduate students in certain major (e.g. psychology), 87% reported anxiety, while 68% reported depression during their training period [2]. This overwhelming stress affects both their daily lives and scientific advancements.

Academic supervisors are supposed to take the primary responsibility for graduate student education as stated in a document issued by the Chinese Ministry of Education. Supervisors serve not only as academic mentors but also as career advisers and moral models. Therefore, the relationship between graduate students and their academic supervisors matters significantly as it could influence their access to resources, academic guidance, professional networks, and career development [3]. However, the power imbalance inherent in the “supervisor full responsibility system”, combined with a lack of third-party oversight, could lead to the abuse of authority, such as excessive criticism, unreasonable demands, or emotional pressure, causing significant psychological stress for students [4]. Additionally, the traditional Chinese cultural value of “respecting teachers and valuing their guidance” encourages students to endure inappropriate behavior silently. Without effective support or accountability, this dynamic would exacerbate the psychological burden on graduate students worsening their mental health. In recent years, strained relationships with academic supervisors have exerted adverse mental health issues among graduate students [4, 5]. Research on the impact of supervisor-student relationship on graduate students’ mental health is still in its nascent stage, rarely explored also as how negative supervisor-student relationships influence graduate students’ mental health. Therefore, this study aims to address this gap by examining the effects and underlying mechanisms of abusive supervision — a form of dysfunctional supervising behaviour — on graduate students’ mental health.

### Abusive supervision

Abusive supervision refers to the continuous hostile behavioral pattern perceived by the subordinates, whether it is verbal or non-verbal. It typically includes teasing and verbal abuse, such as put-downs, blaming and name-calling, excluding physical contact [6]. Abusive supervision is not unusual in the workplace of which the impacts on subordinates and organizations have been extensively studied. Abusive supervision influenced subordinates’ emotions and impaired mental health [7]. For instance, abusive supervision decreases the subjective well-being [8], increased the likelihood of emotional exhaustion [9] and depression [10].

More widely, abusive supervision is an intractable social issue that grows out of formal employment relationships and has been applied to other relationships. Some scholars have introduced abusive supervision into the context of higher education such as the supervisor-student relationship [5] as the relationship mode in the supervisor-student dyads has gradually changed to more subordinate than equal relationships [11]. A simple case is that the students assist in carrying out research projects in exchange for stipend from the supervisor. Yet what weighs heavily is the supervisor’s provision of guidance and resources into the graduate students’ training and his/her decision on whether or not the student could graduate eventually [12]. Students would feel powerless confronting abusive supervision given the unequal power structure inherent in the supervisor-student relationship which inevitablely nourishes abusive supervision. Thus, prolonged unequal pressure and the inability to alter the adverse situation are likely to associated with negative mental health outcomes. This study aims to expand our understanding of whether and how abusive supervision impacts graduate students’ mental health, specifically by exploring the underlying mechanisms of this influence.

### Abusive supervision and graduate students’ mental health

The diathesis-stress model emphasizes negative external environment as the stress of mental health [13]. Abusive supervision as a negative stimulus and an interpersonal stressor, poses adverse outcomes in both short- and long-term mental health. Abusive supervision was proven to be one of the most powerful stressors in workplace, and it exerted detrimental outcomes on mental health [14–16]. Abusive supervision, including supervisor’s hostile attitude and behaviours, means that the supervisors tend to ignore students’ demands for reasonable support or to deprive necessary resources. According to the Conservation of Resources theory, lacking resources might lead to accumulated stress [17] and worsened mental health, such as depression [4]. Also, when the stress of abusive supervision exceeds the students’ coping resources, it would damage one’s mental health as outlined by the transactional model of stress and coping [18].

With the trend of an employment-like supervisor-student relationship, the impact of abusive supervision on graduate students’ mental health could be foreseen. Abusive supervision and other destructive supervisory behaviours by academic advisors affect individuals’ emotional states [19]. Prolonged exposure to high-pressure situations co-occurs with various emotional symptoms, and abusive behaviours exacerbate students’ anxiety and their overall mental health symptom as depression [4]. While direct evidence linking abusive supervision to psychache remains limited, existing research suggest that emotional abuse can induce psychache [20]. Furthermore, studies have shown that reduced perceived social support is associated with increased levels of psychache [21]. Based on theories and previous empirical findings, we hypothesized that abusive supervision was positively correlated with negative mental health outcomes among graduate students (Hypothesis1).

### The mediating roles of autonomy need between abusive supervision and mental health

Autonomy need was one of the three innate fundamental psychological needs for human growth posited by Self-Determination Theory, widely accepted to understand individuals’ motivation and behaviours [22]. Autonomy need refers to the need that individuals feel volitional for their own decisions, actions, and behaviours [23]. The satisfaction of autonomy need is necessary for mental health thus serve as an important promoter of subjective well-being [24] and meaning in life [25]. Young adults whose autonomy need is well satisfied were found to experience better mental health. It was shown that one standard deviation increase in the autonomy need is related to 33% decrease in suicide behaviour and 45% reduction of the odds of suicidal ideation [26].

Self-Determination Theory claims that autonomy-supportive environment, including interpersonal and social contexts facilitate much healthier development of autonomy [22] which then facilitate the motivational process [27]. A supportive supervisory style that fosters autonomy and creativity weighs heavily for graduate students [28, 29] and nourish their mental health. Conversely, abusive supervision, which entails the deprivation of autonomy, could impair mental health among graduate students, giving rise to depression, anxiety, low self-esteem, and somatization [14, 16]. Combining the above theories and empirical research, we hypothesized that autonomy need mediated the relationship between abusive supervision and mental health of graduate students (Hypothesis 2).

### The mediating roles of professional identity between abusive supervision and mental health

Professional identity refers to the degree of acceptance and recognition of the learner’s major, a dynamic psychological experience accompanying the learning process [30] which develops through mentorship, relationship building, self-reflection and professional experiences [31]. Supportive mentoring, which encompasses guidance to discipline frontiers, instruction on research methods and abidance by academic norms, fosters student’s professional identity. The learning context and the role model shape the construction of professional identity and endow meaning to the identity development [32]. Empirical research found that the mentoring from academic supervisors and professional development prospect are two important factors of professional identity [33]. Supervisor, as natural career model for students, plays a vital role in the formation of professional identity [34]. Abusive supervision whereas suppressing and demeaning would surely interfere with student’s academic self-efficacy, professional identity and allegiance [35].

A supportive and comfortable working atmosphere is conducive to positive emotional states and work attitudes of individuals [36]. Hence the affirmation and encouragement from academic supervisors might improve student’s self-efficacy and promote one’s professional identity. On the contrary, suppression and demeaning supervision could impair self-efficacy [37] and professional identity. Moreover, professional identity was verified to be negatively associated with work-related depression and anxiety among nurses [38] and positively correlated to mental health [39], subjective well-being [40] and work-related well-being [41]. Students with stronger professional identity usually achieve better self-actualization and enhanced mental health [42]. Undermined professional identity, in contrast, leave individuals more susceptible to feelings of frustration and helplessness, potentially leading to anxiety and depression and burnout [43]. Therefore, we speculated that professional identity mediated the relationship between abusive supervision and graduate students’ mental health (Hypothesis 3).

### The chain mediation effect of autonomy need and professional identity between the relationship between abusive supervision and mental health

The Conservation of Resources theory posits that individuals strive to obtain, safeguard, and preserve valuable resources, such as autonomy, professional identity, and social support. When these resources are compromised or diminished, individuals would suffer stress and psychological distress. Abusive supervision, characterized by excessive control, belittlement or neglect, directly erodes students’ psychological resources, such as autonomy and support thus, reduces one’s resources. In parallel, the Self-Determination Theory states that the internal urge to fulfill the basic psychological needs, such as autonomy need, intrinsically drives an individual to engage in activities and positively contributes to one’s pursuit of identity [44]. When the need for autonomy is threatened, senses of powerless and passive show up. If this sense of helplessness relates to one’s career development, it could undermine professional identity and further impair one’s mental well-being.

Recent studies have reinforced this perspective. In 2020, scholars conducted an exploration into the formation of professional identity among students and found that both autonomy support and autonomous decision-making exert significant influences on the formation of professional identity [31]. Satisfaction of autonomy need improves individuals’ school engagement [45] and promotes learning involvement [46, 47]. Autonomy was key to cultivating trainee’s growth motivation [31] and contributed to professional identity [48].

A harmonious teacher-student relationship naturally satisfies students’ autonomy needs, thus strengthens their professional identity. In the training of medical residents, autonomy granted by the supervisor facilitated resident’s responsibility and engagement, whereas lack of autonomy resulted in their disengagement [31]. The perceived teacher’s emotional support could facilitate student’s autonomy satisfaction so as to enhance learning engagement [49]. The autonomy support from the coaches predicted students’ self-esteem and identity [50]. Notably, a significant positive correlation exists between professional identity and mental health [43].

Despite the scarcity of research exploring the negative impacts of abusive supervision on mental health from the perspective of students’ professional development, based on the theoretical frameworks of Conservation of Resources Theory, Self-Determination Theory and Professional Identity Formation, we propose the last hypothesis. Abusive supervision that lacks autonomy support would undermine students’ autonomy needs and diminish their professional identity. Consequently, students with weaker professional identities may find themselves vulnerable to feelings of frustration and helplessness at the cost their mental well-being. The autonomy need and professional identity were proposed to play chain mediating roles in abusive supervision and mental health (Hypothesis 4).

### Present study

Our study examined the impacts abusive supervision had on graduate students’ mental health. Grounded in the Conservation of Resources Theory and Self-Determination Theory, a theoretically hypothesized chain mediation model was proposed in the study, encompassing the following hypotheses. First, perceived abusive supervision positively correlates to mental health among graduate students. Second, abusive supervision impairs students’ autonomy need as well as professional identity significantly thereby increases mental health. That is autonomy need and professional identity respectively mediates the link between abusive supervision and mental health. Third, autonomy need and professional identity play the chain mediating roles between abusive supervision and mental health.

## Materials and methods

### Participants and sampling

A cross-sectional online survey was conducted among graduate students from five Chinese universities (Xiamen University, Zhejiang University, University of International Business and Economics [Beijing], Northwest University, and Hebei Agricultural University), using convenience sampling and snowball sampling methods. Graduate students who have spent more than one semester with their supervisors meet the requirements of our study, and students with diagnosis of schizophrenia were excluded. From the initial pool of 250 respondents, 233 participants (age range = 22–33 years; M age = 24.57, SD = 1.99;61.8% identified as female) met all inclusion criteria and comprised the final analytical sample, yielding a 93.2% valid response rate. In terms of academic disciplines, 68.1% were from natural sciences, 30.4% from humanities and social sciences, and 1.5% from other fields. The purpose and procedure of the study was firstly introduced and then the online written consents were signed before the survey. The study was approved by the Research Ethics Review Committee of Beijing Normal University.

### Measures

***Perceived abusive supervision*** The 10-item unidimensional scale was adapted from Abusive Supervision scale [51] to measure continuous hostile verbal or nonverbal supervisory behaviours perceived by graduate students in their relationship with the supervisors. It was assessed with 5-point from 1 (never) to 5 (always). Sample items are “my supervisor ignores or gives me the silent treatment” and “my supervisor tells me my thoughts or feelings are stupid”. The internal consistency in the study was 0.896. The structure validity was acceptable, with fit indices of *CFI* = 0.930, *TLI* = 0.898, *RMSEA* = 0.103, *SRMR* = 0.051).

***Autonomy need*** The 8-item unidimensional subscale of the Basic Psychological Need Satisfaction and Frustration Scale [52] was used to measure the satisfaction of autonomy need. It was a 5-point Likert scale ranging from 1(completely disagree) to 5 (completely agree). Higher scores indicated more full satisfaction of the autonomy need. Example items included “In my life, I can do whatever I want when I want” and “I feel my choices express my true self”. The Cronbach’s alpha coefficient of the subscale in our study was 0.794. As indicated by the fit indices, the structure validity was good (*CFI* = 0.997, *TLI* = 0.996, *RMSEA* = 0.030, *SRMR* = 0.015).

***Professional identity*** The professional identity scale contained 23 items [53] under four dimensions: fitness, cognition, behaviour and emotionality. Items such as “I am willing to engage in work related to my major” and “My major can reflect my specialty” were rated ranging from 1 (completely disagree) to 5 (completely agree). Higher scores indicated stronger professional identity. The internal consistency coefficients of the four dimensions in this study respectively were 0.808, 0.901, 0.851 and 0.838, respectively. The whole scale showed good reliability (Cronbach’s α = 0.945) and acceptable structural validity (*CFI* = 0.888, *TLI* = 0.869, *RMSEA* = 0.097, *SRMR* = 0.089).

***Mental health outcomes*** The outcome variable “mental health outcomes” was a latent variable constructed on three components: anxiety symptoms, depressive symptoms and psychological pain (psychache). The 7-item seven-item Generalized Anxiety Disorder Scale was used in our study. Participants rated the frequency of symptoms over the past 2 weeks on a four-point scale from 0 (not at all) to 3 (nearly every day). An anxiety score was calculated by summing up the seven items, with a higher score indicating a greater severity of anxiety symptoms. And the structure validity was acceptable (*CFI* = 0.986, *TLI* = 0.974, *RMSEA* = 0.079, *SRMR* = 0.020). The nine-item self-report Patient Health Questionnaire (PHQ-9) was used to assess the degree of depressive symptoms in the latest two weeks, for example “little interest or pleasure in doing things” and “Feeling down, depressed or hopeless”. The PHQ-9 was rated on a 4-point Likert scale ranging from 0 (not at all) to 3 (nearly every day). The severity of depressive symptoms was calculated using the sum of all nine items. The indices for this scale also indicated adequate validity (*CFI* = 0.936, *TLI* = 0.908, *RMSEA* = 0.108, *SRMR* = 0.041). To assess psychological pain (psychache), we adopted the Psychache Scale, a 13-item measure designed to capture the intensity of inner suffering. Samples like “I seem to ache inside” and “My pain is making me fall apart” were responded on a 5-point Likert rating. The Psychache Scale demonstrated satisfactory model fit (*CFI* = 0.921, *TLI* = 0.899, *RMSEA* = 0.125, *SRMR* = 0.045). and the internal consistency of the three scales was 0.921, 0.911 and 0.956 respectively in this study.

### Data analysis

Descriptive statistics and correlational analysis were reported. Given that the continuous variables were all non-normal, Spearman correlation was used to analyze the correlation. The mediation model hypothesized was tested by using structural equation modeling (*SEM*). Model fit was assessed through established indices [54], including Normed *χ*^*2*^ and the following goodness-of-fit indices thresholds: 0.90 or above for the parameters Tucker-Lewis Index (*TLI*) and Comparative Fit Index (*CFI*), 0.08 or below for Standardized Root Mean Residual (*SRMR*) and Root Mean Square Error of Approximation (*RMSEA*). In order to examine the significance of the mediation effect, the bias-corrected bootstrapping method with a 95% confidence interval (*CI*) from 5000 samples was computed. All the above data analyses were conducted in SPSS version 23.0 and Mplus version 8.0.

## Result

### Common method bias test

To assess potential common method biases, the Unmeasured Latent Method Construct (ULMC) approach was employed. We incorporated the common method factor into the original model, and built a five-factor model to test for the potential common method variance. The comparison revealed minimal differences in model fit indices between these configurations (*ΔCFI* = 0.01, *ΔTLI* = 0.02, *ΔRMSEA* = 0.01, *ΔSRMR* = 0.01), and the change value of each fit index did not exceed the empirical value of 0.02, suggesting no substantial common method bias in the measured data [55].

### Background characteristics and covariates

The descriptive statistics and the correlation matrix of the variables are presented in Table 1. The Spearman’s correlation test showed there were correlations between abusive supervision, the autonomy need, the four dimensions of professional identity and three indicators of mental health. The pairwise correlations of the three indicators of mental health (anxiety, depression, psychache) respective were 0.751, 0.691 and 0.730 (*p* < 0.01). The correlation between anxiety and abusive supervision (*r* = 0.310, *p* < 0.01), autonomy need (*r* = -0.306, *p* < 0.01) and professional identity (*r* = -0.249 to -0.377, *p* < 0.01) were all significant. Depression was significantly related to abusive supervision (*r* = 0.396, *p* < 0.01), autonomy need (*r* = -0.448, *p* < 0.01) and professional identity (*r* = -0.20 to -0.346, *p* < 0.01). Psychache was significantly associated with abusive supervision (*r* = 0.343, *p* < 0.01), autonomy need (*r* = -0.402, *p* < 0.01) and professional identity (*r* = -0.230 to -0.426, *p* < 0.01).

**Table 1:** Demographic characteristics and correlation between main variables

		M ± SD	1	2	3	4	5a	5b	5c	5d	6a	6b	6c
1 Gender		0.38 ± 0.487	1.000										
2 Age		24.60 ± 2.06	0.083	1.000									
3 Abusive supervision		1.75 ± 0.76	0.070	0.150^*^	1.000								
4 Autonomy need		3.03 ± 0.69	0.092	-0.057	-0.207^**^	1.000							
5 Professional identity	5a Fitness	3.97 ± 0.52	0.119	0.104	-0.213^**^	0.184^**^	1.000						
	5b Cognition	3.56 ± 0.80	0.007	0.052	-0.275^**^	0.329^**^	0.400^**^	1.000					
	5c Behaviour	3.41 ± 0.77	0.059	0.126	-0.302^**^	0.312^**^	0.444^**^	0.793^**^	1.000				
	5d Emotionality	3.35 ± 0.76	0.086	0.173^**^	-0.212^**^	0.311^**^	0.474^**^	0.700^**^	0.809^**^	1.000			
6 Mental health	6a Anxiety	1.92 ± 0.66	-0.028	-0.012	0.310^**^	-0.306^**^	-0.249^**^	-0.337^**^	-0.365^**^	-0.377^**^	1.000		
	6b Depression	1.80 ± 0.65	-0.035	0.009	0.396^**^	-0.448^**^	-0.200^**^	-0.300^**^	-0.346^**^	-0.343^**^	0.751^**^	1.000	
	6c Psychache	1.85 ± 0.81	-0.159^*^	0.006	0.343^**^	-0.402^**^	-0.230^**^	-0.311^**^	-0.385^**^	-0.426^**^	0.691^**^	0.730^**^	1

Note. *Gender was coded as 1 = Male*,* 0 = Female.* *p < 0.05, **p < 0.01

### Mediating effects test

Taking abusive supervision as the independent variable, the latent variable mental health as the dependent with gender, age and discipline as covariates, the hypothesized model was examined by SEM. Results showed that the model fit well (*χ*^*2*^*/df* = 2.625, *TLI* = 0.955, *CFI* = 0.976, *SRMR* = 0.027, *RMSEA* = 0.084). Abusive supervision had significant main effect on mental health symptoms after controlling for covariates (*β* = 0.390, 95% CI = [0.256, 0.523], *p* < 0.001). The results supported that the higher level of abusive supervision the graduate students perceived, the more negative mental health they were likely to report.

We systematically evaluated model fit across baseline models, partial models, and full models, while examining single mediation, parallel mediation, and chain mediation effects, and models are described in details in Table 2. The findings demonstrate that the chain mediation model provides a better statistical fit compared to either single mediation models or the parallel mediation model in terms of overall model fit.

**Table 2:** Model fit across different frameworks

	χ^2^/df	TLI	CFI	SRMR	RMSEA
Baseline model	2.625	0.955	0.976	0.027	0.084
Single mediation model (autonomy need as the mediation)	2.542	0.943	0.966	0.035	0.081
Single mediation model (profession identity as the mediation)	2.965	0.922	0.941	0.080	0.092
Parallel mediation model	2.739	0.919	0.940	0.078	0.086
Chain mediation model	2.233	0.938	0.956	0.052	0.075

**Fig. 1:**
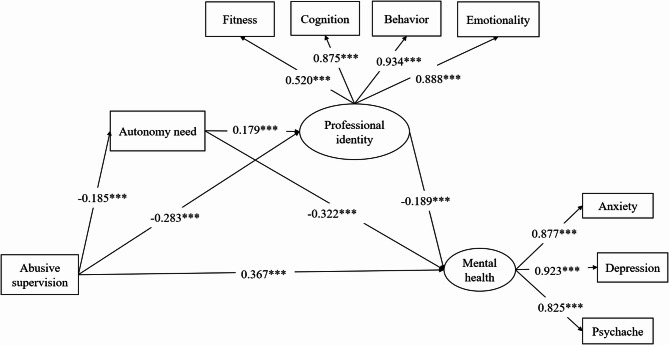
Abusive supervision and the mental health among graduate students: the chain mediation effect of autonomy need and professional identity. ^*^p < 0.05, ^**^p < 0.01, ^***^ *p* < 0.001

To test the chain mediation model, we conducted SEM using abusive supervision as predictor, autonomy need and the latent variable professional identity as the chain mediators on mental health with three covariates being controlled (gender, age and discipline). The model fits the data well (*χ*^*2*^*/df* = 2.233, *TLI* = 0.938, *CFI* = 0.956, *SRMR* = 0.052, *RMSEA* = 0.075) and the path coefficients are shown in Fig. 1.

The perceived abusive supervision significantly exacerbated negative mental health outcomes (*β* = 0.367, 95% CI = [0.180, 0.423], *p* < 0.001) while decreased the autonomy need (*β* = -0.185, 95% CI = [-0.270, -0.066], *p* = 0.001) of the graduate students. Besides, autonomy need reduced the negative mental health symptoms of the graduate students (*β* = -0.322, 95% CI = [-0.371, -0.181], *p* < 0.001). The results suggested that autonomy need functioned as a mediator between perceived abusive supervision and mental health. The mediating pathway had the effect size of 0.060 (*p =* 0.004) as presented in the Table 3 with the 95% confidence interval being [0.019, 0.100].

Abusive supervision negatively affected professional identity (*β* = -0.283, 95% CI = [-0.312, -0.172], *p* < 0.001), and professional identity was negatively correlated with mental health (*β* = -0.189, 95% CI = [-0.637, -0.179], *p <* 0.001). This indicated that professional identity mediated abusive supervision’s impact on mental health with the mediating effect size as 0.054, 95% CI = [0.017, 0.090], *p* = 0.004, suggesting a significant mediation effect. In addition, autonomy need positively predicted professional identity (*β* = 0.179, 95% CI = [0.052, 0.191], *p* < 0.001). The abusive supervision affected autonomy need, professional identity, and furthermore affected the mental health of graduate students. The chain mediating effect is significant, effect size being 0.006, 95% CI = [0.001, 0.011], *p* = 0.028, and the total indirect effect size of the chain mediation model is 0.119, 95% CI = [0.066, 0.173], *p* < 0.001, accounting for 24.44% of overall effect size. The results of Bootstrap test of the mediating effects were shown in Table 3.

**Table 3:** Bootstrap test and effect size of mediating effects

Effects	Pathways	*β*	*p* value	95% confidence interval	
				Lower limit	Upper limit
Direct effect	AS -MH	0.367	< 0.001	0.246	0.487
	AS - AN- MH	0.060	0.004	0.019	0.100
Mediation paths	AS - PI - MH	0.054	0.004	0.017	0.090
	AS- AN- PI -MH	0.006	0.028	0.001	0.011
Total indirect effect		0.119	< 0.001	0.066	0.173
Total effect		0.487	< 0.001	0.371	0.602

Note: AS: Abusive Supervision; AN: autonomy need; PI: Professional Identity; MH: Mental health

In conclusion, the perceived abusive supervision influenced the mental health of graduate students both directly and indirectly through mediating effect of autonomy need and professional identity respectively and through the chain mediation of them.

## Discussion

There are four main findings in our study. Most importantly, it verified that perceived abusive supervision negatively correlated with the mental health level of graduate students. The result beared out and supplemented the conclusion that abusive supervision was highly associated with adverse mental health outcomes [17], exacerbated anxiety, depression [14] and other mental health issues [5]. The finding is in line with Affective Events Theory that a hostile working environment directly induces the negative emotions and emotional exhaustion. We are afraid that the negative impacts may be even more severe for graduate students, as some studies have found that abusive supervision might increase suicidal risk [5, 16]. The arrested flight model [56] points out that when one is trapped in difficult situations that he could neither face nor escape from, a sharp rise in feeling of despair occurs. In the higher education context, supervisor alternation was a highly costly and complicated process and it would be quite suffering and difficult for graduate students to break off from an abusive relationship. Stuck in this impasse, aggressive or irrational behaviours of the supervisee like self-destructive and suicidal attempts are not impossible [13].

Secondly, autonomy need played a mediation role between the perceived abusive supervision and mental health. Supportive relationship would promote self-determined motivation [22] and benefit mental health outcomes through the autonomy need satisfaction [50]. In contrast, when confronted with abusive supervision, students are more likely to accommodate the supervisors’ expectations to prevent further harm. Previous studies found abusive supervision positively correlated to employees’ surface acting and negatively correlated to deep acting [57] and individuals were more likely to cyberloaf in circumstance of abusive supervision [58] which implied that abusive supervision impede individuals’ intrinsic motivation and impair autonomy [15]. Thus, graduate students had to cope with abusive supervision at the cost of their basic need of autonomy.

In addition, autonomy was crucial for decisions making, confidence building and responsibility taking for one’s work [31]. An important task during the graduate training phase is to actively create meaningful and original knowledge, where intrinsic motivation, autonomy and innovation are highly requisite. However, long-term abusive environment would significantly reduce students’ intrinsic motivated behaviour and hinder one’s autonomy satisfaction. The conflicts between the innovation requirement and the reduction of autonomous behaviours caused by abusive supervision would engender enormous pressure and even render despair. Meanwhile, unmet autonomy need also has been shown to mediate the negative life events and mental health [24]. Therefore, from a practical perspective, supervisors should acknowledge the importance of student’s autonomous exploration to their mental health [14, 59].

Furthermore, professional identity was verified to mediate the relationship between perceived abusive supervision and mental health. This finding validated the social identity theory in that positive identity was gained and maintained through continuous positive interactions with the supervisor and that professional identity stimulated students’ positive emotions during professional learning process [60]. Studies based on the Social Cognitive Career Theory confirmed that mentorship, a kind of contextual factors facilitated or impeded the profession progress and career exploration intents for the graduate students [61], could be instrumental in the formation and maturation of professional identities [62]. A favorable supervisor provides role model for professional endeavor and ideals [31]. On the contrary, the frequent abusive supervision frustrates students’ academic enthusiasm, reduces their academic involvement, thus degrades their professional identity. Our study corroborated the correlation between professional identity and negative mental health symptoms, including anxiety, depression, and psychache.

Notably, perceived abusive supervision affected mental health through the chain mediating effect of autonomy need and professional identity. In previous studies, autonomy fully mediated the relationship between faculty support and major satisfaction [63] and it also mediated the influence of maternal psychological control in academic self-concept of Chinese adolescents [64]. Abusive supervisors tend to limit students’ autonomy, express distrust toward them and impair their professional confidence [61]. In an abusive supervisory environment, the unmet autonomy need could be associated with lower professional satisfaction and involvement, and linked to weaker professional identity and poorer mental health [40, 43].

### Contributions

This study innovatively extended the concept of “abusive supervision” traditionally examined in workplace settings, to the academic context, focusing on supervisor-student relationships. This approach deepened theoretical understanding of how abusive supervision operates in educational contexts, offering a novel perspective on power and control dynamics in these interactions. It offered practical insights for addressing mental health challenges in high-pressure educational settings, particularly within hierarchical student-supervisor relationship. Furthermore, by integrating Conservation of Resources Theory and Self-Determination Theory, this study provided a robust theoretical framework for understanding the impacts of abusive supervision on graduate students’ mental health which in return enriched both the two theories on promoting psychological resources and self-determination. In all, this research significantly contributed to the literature on abusive supervision, academic development and mental health.

The results here led to several implications. This is the first time that the impacts of abusive supervision on graduate students’ mental health have been examined from the perspective of professional growth. Our research found the possible mechanism behind the mental health issue caused by the poor supervisor-student relationship and provided an empirical basis for practical work in colleges and universities. It warned us to recognize and handle cautiously the harmful influence of abusive supervision which would otherwise cause severe damage to the mental health of graduate students [5]. Meanwhile, the results of the study also suggested that education departments and schools should incorporate abusive supervision into the supervisor evaluation to effectively regulate supervisors’ ethics and morale.

Additionally, our study confirmed that autonomy need, professional identity, and their chained mediating effect serve as mechanisms to understand the relationship between abusive supervision and mental health. On one hand, this result suggested an important role of supportive supervision, not only for academic development but also for students’ mental health. A supportive supervisor-student relationship would fulfill the basic need for autonomy, facilitate students’ professional identity and mitigate adverse mental health outcomes. Supervisors are suggested to discuss with students more democratically and to encourage students to fully articulate their professional ideas, but not to impose on them. Supervisors are encouraged to be role models of students’ professional growth, influencing students by precept and example. A positive and integral career role model could improve the enthusiasm of the graduates for academic learning, increase their positive emotional experience in professional study, and enhance their professional identity. On the other hand, the results also offered valuable insights into practices for improving mental health. Mental health workers in universities could help students to cope with distress by promoting their experiential autonomy, for example, supporting them to retain life choices to themselves. At the same time, these results provided empirical evidence for the Self-Determination Theory and inspired preventive measures to promote autonomy and professional identity in order to decrease negative mental health outcomes.

### Limitations and future directions

This study was a preliminary attempt targeting the impacts of abusive supervision on graduate students’ mental health. The cross-sectional design precluded causal inferences due to single-timepoint data collection. Future research should employ longitudinal methods to establish the temporal sequencing of abusive supervision effects and multi-wave designs to examine mediation processes, thereby addressing this limitation. Second, the sample size was slightly below the required threshold, which may reduce the statistical power or generalizability of the results. Further studies with larger and more diverse samples are recommended to validate these conclusions. Furthermore, the reliance on student self-reports may not fully reflect actual supervisory behaviors, and single-source evaluations could introduce bias due to differing teacher-student perspectives [57]. Future studies should combine multi-source data (e.g., supervisor self-reports, third-party observations) and behavioral coding to objectively assess supervisory interactions.

While the findings provided initial insights, the indirect effect accounted for a relatively modest proportion (24.44%), suggesting that other factors and mechanisms may play significant roles. For instance, individual differences, such as self-efficacy, and institutional support systems, including mental health resources and peer support networks, could further explain the varying impacts of abusive supervision. Additionally, the unique dynamics of the Chinese academic environment, such as the hierarchical nature of supervisor-student relationships and the cultural pressure of “respecting teachers and valuing their guidance” may amplify the effects of abusive supervision. Future research could further validate the potential differential impacts of abusive supervision across different cultural contexts, and larger, more diverse samples are needed to enhance the generalizability of the findings.

As research on abusive supervision in supervisor-student relationships is still emerging, addressing these gaps can deepen our understanding of its mechanisms and effects on graduate students’ mental health, paving the way for targeted interventions and support.

## Conclusions

This study advanced empirical understanding of the potential mechanisms through which abusive supervision influenced mental health of the graduate students. The results confirmed both the direct and indirect effects of abusive supervision on mental health, with the mediation effects of autonomy need and professional identity, as well as their combined chain mediation effect. Abusive supervision contributes to mental health problems by potentially impeding the autonomy need satisfaction and corroding the professional identity of graduate students. Empirical validation and implications for mental health education and intervention were discussed.

## Supplementary Information

Below is the link to the electronic supplementary material.

Supplementary Material 1

## Data Availability

No datasets were generated or analysed during the current study.

## Abbreviations

AS: Abusive supervision
AN: Autonomy need
PI: Professional identity
MH: Mental health
SEM: Structural equation modeling
ULMC: Unmeasured latent method construct
CI: Confidence interval
