# Supplementary material for: Perceived abusive supervision and mental health among Chinese graduate students: the chain mediating roles of autonomy need and professional identity
Source: BMC Psychol. 2025 Aug 23;13:959. doi: 10.1186/s40359-025-03324-5 (PMC12374291; doi:10.1186/s40359-025-03324-5)
Supplement: Supplementary file 1 — Supplementary Material 1 [file 40359_2025_3324_MOESM1_ESM.pdf]

Ethics Review No.	20221201120

Faculty of Psychology, Beijing Normal University

Ethics Review Committee Scientific Research Project Review Approval Document

**Project Name**	The impact of Supervisor-supervisee relationship on graduate students' mental health				
**Principal Researcher**	Qiao Zhihong	**Organization**	Beijing Normal University	**Responsibility**	☑In charge<br>☐ Participate in
**Review Comments**	☑Approved ☐Approved with necessary modifications<br>☐Disapproved ☐Terminate or suspend the approved experiment				

Project Description：

This study uses semi-structured interviews, online questionaries and empirical sampling to observe the changes of the graduate student's daily mental health states. Meanwhile, it designs a group intervention approach to improve the positive psychological qualities of graduate students, in order to provide empirical support for improving the relationship between supervisors and students and the mental health of graduate students.

This research process does not involve drugs or narcotics and poses no harm to the human body.

Please comply with the following terms and conditions In the process of conducting this research project :

1. Voluntary principle: Researchers inform participants or other individuals of the potential risks and benefits, and participants voluntarily participate (by signing an informed consent form). Participants have the right to withdraw from the experiment at any stage without discrimination or retaliation, and their medical treatment and rights will not be affected.；
2. Confidentiality principle: All personal information related to participation in the experiment and obtained during the experiment is confidential. Only higher-level administrative departments and ethics review committees may access the information of participants in the experiment in accordance with regulations.；
3. Safety principle: The interests of the participants should be given priority over scientific and social considerations. Any serious adverse events occurring during the experiment should be reported to the ethics review committee in a timely manner；
4. Compensation principle: When damage related to the experiment occurs, participants can receive treatment and appropriate compensation；
5. Other internationally accepted principles of academic ethics review。

Notes: Researchers are requested to conduct research strictly in accordance with the approved research protocol. Any modifications must be submitted to the Ethics Review Committee for discussion and approval。

Ethics and Human Safety Commitee, Faculty of Psychology, Beijing Normal University

Principal Researcher Signature: [illegible]

Research Direction Commitee Signature: [illegible]

Chair of the Ethics Commitee Signature: [illegible]

November 25, 2022
